# Promoting healthy sleep in 0-2-year-old infants: a study protocol for the development and mixed method evaluation of a sleep health program tailored to Dutch youth healthcare regions

**DOI:** 10.1186/s12889-024-19258-3

**Published:** 2024-07-16

**Authors:** MPW van de Sande, SMPL Gerards, MP L’Hoir, A Gabrio, RP Reijs, I Tissen, SW van Dam, FHGY Alberts, RM Meertens

**Affiliations:** 1 Department of Health Promotion, NUTRIM School of Nutrition and Translational Research in Metabolism, Maastricht University, P.O. Box 616, Maastricht, 6200 MD The Netherlands; 2 Department of Global Nutrition, Division of Human Nutrition and Health, Wageningen University and Research, P.O. Box 17, Wageningen, 6700 AA The Netherlands; 3 Public Health Service North-East-Gelderland, P.O. Box 3, Zutphen, 7200 AA The Netherlands; 4 Department of Methodology and Statistics, CAPHRI Care and Public Health Research Institute, Maastricht University, P.O. Box 616, Maastricht, 6200 MD The Netherlands; 5 Department of Social Medicine, CAPHRI Care and Public Health Research Institute, Maastricht University, P.O. Box 616, Maastricht, 6200 MD The Netherlands; 6 Department of Youth Health Care, Public Health Service South Limburg, P.O. box 33, Heerlen, 6400 AA The Netherlands; 7 Public Health Service Limburg-North, P.O. box 1150, Venlo, 5900 BD The Netherlands; 8 Public Health Service Brabant-Southeast, P.O. box 8684, KR Eindhoven, 5605 The Netherlands

**Keywords:** Intervention, Behavioural intervention, Sleep problems, Prevention, Child Development, Primary Health Care, Health Education, Behaviour Therapy, Sleep Wake disorders, Netherlands

## Abstract

**Background:**

Sleep problems are common among infants and can have a serious impact on the health and wellbeing of both child and parents. To sustainably promote infant sleep on a population level, it is necessary to develop evidence-based programs that can be implemented on a large scale. The Youth Health Care setting, with its focus on prevention, child health promotion and services widely available for parents, can be a suitable setting to do so. Currently however, sleep health promotion in this setting seems to be suboptimal. To promote healthy infant sleep on a population level, programs need to be accessible and comprehensible for all parents, including parents with limited (health) literacy. Therefore, this study aims to develop, implement and evaluate a program called ‘Sleep on number 1’, that is tailored to Dutch Youth Health Care, to sustainably promote healthy sleep in 0-2-year-old infants.

**Methods:**

The program was developed based on co-creation with parents and Youth Health Care professionals, evidence-based behaviour change theories and sleep health promotion methods. Program effectiveness is investigated with a quasi-experimental study design comparing the program group with the care as usual control group. Participants consist of parents of 0-2-year-old children. Primary outcome is infant sleep quality at the age of 10 weeks and 6, 9, 14 and 24 months, measured with a sleep diary. The primary data analysis focuses on night awakenings at 9 months. Secondary outcomes focus on parental behaviour regarding infant sleep, related behavioural determinants and parental satisfaction with Youth Health Care sleep advice. Program effectiveness is analysed using a linear mixed-model in case of data clustering, and an independent samples T-test or linear regression in case no substantial clustering effects are found. A mixed methods process evaluation is performed with parents and Youth Health Care professionals, assessing program reach, adoption, implementation, maintenance and working mechanisms.

**Discussion:**

The ‘Sleep on number 1’ program is an evidence-based sleep health program for 0-2-year-old children, tailored to Dutch Youth Health Care. If effective, this program has the potential to improve infant sleep on a population level.

**Trial registration:**

ISRCTN, ISRCTN27246394, registered on 10/03/2023. https://www.isrctn.com/ISRCTN27246394.

**Supplementary Information:**

The online version contains supplementary material available at 10.1186/s12889-024-19258-3.

## Background

Sleep problems are common amongst infants. According to international studies, approximately 20–30% of infants and toddlers experience sleep problems such as delayed sleep onset, frequent nighttime awakenings and early awakenings [1–4]. Moreover, many parents report having difficulties with managing their infant’s sleep, even when their child’s sleep appears to be normal [5–7]. Since infant sleep differs significantly from adult sleep, often includes night awakenings and is subject to many changes during infant development, it can be a challenge for parents to adequately manage infant sleep behaviour [2, 8]. Consequently, infant sleep is an issue parents frequently seek help for from healthcare professionals [6, 9–11].

When infant sleep problems arise, they can seriously impede the child’s development by its various negative outcomes such as behavioural and cognitive problems, social-emotional problems and a higher risk on overweight and obesity [12–15]. Furthermore, sleep problems at an early age can persist into childhood and may become chronic [3, 16–18]. Infant sleep problems can have serious consequences for parents as well. They have been associated with parental depression, increased parental stress, reduced parental sleep quality and quantity and worse overall parental health [1, 19–22]. Interestingly, research indicates a reciprocal relationship between infant sleep and parenting effectiveness [5, 10, 23]. Due to this reciprocity, infant sleep problems may result in a vicious circle wherein parental fatigue reduces parent’s ability to adequately manage their child’s sleep, leading to a continuation of the sleep problem [5, 10, 23].

Many factors influence infant sleep and many factors determine whether or not infant sleep becomes problematic. To describe these factors, Sadeh and Anders developed the transactional systems model of sleep-wake regulation in infants [23, 24]. This model stresses the importance of the direct relationship between infant sleep and parent-infant interactions [23, 24]. Behaviours such as reduced parental limit setting as well as a high level of assistance in the child falling asleep, such as soothing the child to sleep, are related to the development of sleeping problems in young children. Such behaviours may interfere with the development of self-regulation skills in the child, which are necessary for the child to be able to self-soothe and fall asleep independently [5, 10, 23, 24]. Infant sleep is also subject to significant income-related health disparities: children with a lower Socioeconomic Position (SEP) generally have a shorter sleep duration, more sleep problems and poorer sleep habits in comparison with children of higher SEP [25–28]. Consequently, addressing infant sleep and related parent-infant interactions seems to be a crucial step in promoting healthy infant development, providing support for parents and reducing health disparities.

Various sleep health promotion programs that target these parent-infant interactions in healthy infant populations have been developed and evaluated [1, 29–33]. Such programs often comprise of educational or behavioural elements or a combination of the two. In general, the purpose of educational programs is to prevent sleep problems by providing parents with information about normal sleep and crying patterns, infant cues and strategies to promote infant self-settling (e.g. putting the child to bed drowsy but awake), but can also include education on behavioural strategies [30, 32]. Behavioural programs often comprise of elements such as a consistent sleep schedule, bedtime routines and sleep management techniques focused on removing the reinforcement of the problematic sleep behaviour, by using controlled crying strategies [1, 29–33].

Several systematic reviews indicate that such educational, behavioural or combined strategies are effective in promoting parental behaviours that improve infant sleep, such as setting regular bed and waking times, leaving the infant to self-soothe to sleep and setting adequate limits [30, 32]. Furthermore, such strategies can increase parental knowledge, reduce parental doubts and improve parental comfort in the management of infant sleep. Regarding infant sleep outcomes, such programs lead to fewer nocturnal awakenings, increased nocturnal sleep duration, better sleep quality and parents experiencing the sleep problem as less severe, both in breastfed and formula fed infants [29, 30, 32, 33]. Lastly, with respect to parental health, programs can reduce parental depressed mood and fatigue and can enhance parental sleep [30, 33]. Thus, both infants and parents can benefit from the use of such programs.

To sustainably promote healthy infant sleep on a population level, it is necessary that such programs are developed and implemented on a large scale. One suitable setting to do so in the Netherlands, could be the Youth Health Care (YHC) setting, sometimes also known as the Paediatric Primary Care setting [27, 30, 34, 35]. Examples of this setting in other countries are amongst others Well-Child Care in the United States and Child Health Care in Sweden [36]. The YHC setting can be beneficial for infant sleep health promotion for several reasons. First of all, infant sleep is a theme that is considered important to be addressed in YHC and is a topic of concern for many parents, so infant sleep programs would align with the aims and context of this setting [27, 30, 34, 37]. Secondly, programs in this setting have the potential to reach many families, including families from diverse backgrounds, which could ultimately reduce the prevalence of sleep related health disparities [28, 30, 34, 35, 38]. Lastly, with a service structure consisting of relatively frequent consultations in the infant’s first years of life, YHC professionals can provide parental support continuously and directly from the start [30, 34, 39, 40].

Despite this, several studies indicate that the current screening, prevention and treatment of infant sleep problems in YHC is suboptimal [27, 34, 41, 42]. During their education, YHC professionals, usually comprising of physicians and nurses, often lack training on the management of infant sleep problems, leading to limited knowledge and self-efficacy on how to adequately help parents with these problems [27, 41–43]. Furthermore, professionals often experience time constraints and competing demands, whilst parents have a need for more reliable sleep information and support [27, 30, 35, 43].

To address these issues and successfully and sustainably implement the program, it is crucial to tailor the program to the local YHC context [44–46]. To begin with, it is important to take into account parents’ needs, wishes, barriers and facilitators regarding infant sleep management, for they are the main program target group and key agent in promoting healthy sleep in their child [44, 47]. Secondly, to make the program accessible for a large range of parents, it is necessary that program components are suitable and comprehensible for parents of various educational levels, (health) literacy levels and cultural backgrounds [28, 48]. Finally, it is important to involve YHC professionals in program development, for they are an important target group as well as a crucial stakeholder in program implementation [27, 45]. Therefore, program development in the YHC setting asks for cooperation between the research team and relevant stakeholders such as parents and YHC professionals, instead of a top-down approach [35, 44–46].

Taken together, there is a need for a new sleep health program that focuses on integration in the YHC setting and accessibility to a large audience, to effectively promote healthy infant sleep on a large scale. This study aims to address this gap by developing, implementing and evaluating a program that is tailored to the Dutch YHC setting. Primary focus is to develop a program in co-creation with YHC professionals and parents in two YHC regions in the Netherlands. A quasi-experimental controlled study will be conducted to investigate whether the program leads to better sleep (i.e. longer total sleep duration across 24 h, less time to sleep onset, less nighttime awakenings and less sleeping problems) in infants at the age of 10 weeks and 6, 9, 14 and 24 months, when compared with Dutch YHC regions that provide care as usual. A second aim is to evaluate program effectiveness on parental and intermediary outcomes, such as parental behaviour regarding infant sleep management and parental satisfaction with YHC sleep advice. Lastly, a process evaluation will be executed to gain insight in program reach, adoption, implementation, maintenance and working mechanisms. This protocol paper describes the program development process and study design for evaluation of the study outcomes.

## Methods

### Study design

Based on the RE-AIM model (Reach, Effectiveness, Adoption, Implementation and Maintenance) a mixed methods study design will be used for the evaluation of the ‘Sleep on number 1’ program [49]. To analyse program effectiveness, a quasi-experimental controlled (post-test only) study with one program group and one care as usual control group will be executed. Additionally, a mixed methods process evaluation will be conducted to get insight in program reach, adoption, implementation, maintenance and working mechanisms. This trial was prospectively registered (ISRCTN, ISRCTN27246394). This paper is written according to the Standard Protocol Items: Recommendations for Intervention Trials (SPIRIT) guidelines [50]. The items from the SPIRIT checklist are completed in an additional file [see additional file 1].

### Study setting

This study takes place in the Dutch YHC setting for children aged 0–4 years. In the Netherlands, YHC is a nation-wide service providing preventative care for children aged 0–18 years and includes vaccinations, monitoring of infant growth and consultations for parents regarding infant development, health and parenting [51, 52]. It is available for all parents in the Netherlands, reaches up to 95% of young children and offers frequent consultations in the infant’s first four years of life, with on average nine consultations in the first year [40, 51, 52]. YHC is organised in regions, with parents receiving preventative care from the YHC centre of the region the infant is registered in. The program of this study is developed and implemented in two YHC regions (program region 1 and program region 2) in the South of the Netherlands. Program allocation was not random, for the program regions were chosen because of practical reasons and willingness to develop and implement a program in their organisation. The care as usual control group is formed by all YHC regions in the Netherlands that were not exposed to the program nor any of the program elements and provided usual care to parents. In the Netherlands, all YHC regions should adhere to the Dutch YHC infant sleep guidelines and the Dutch National Professional Framework for their infant sleep care [51, 53, 54]. Therefore, although there may be some regional variations in how these guidelines are applied, the standard of infant sleep care is expected to be similar across regions in the control group. Researchers, parents and YHC professionals were not blinded to the program.

### Participants

This study received formal ethics approval from the Faculty of Health, Medicine and Life sciences Research Ethics Committee Maastricht University (FHML-REC/2022/009/Addendum01_22). Participants eligible for the study consist of adult parents (or primary caregivers of infants) whose infant is registered in a YHC region included in either the program or control group. At the time of inclusion, the newborn should be 0–10 weeks old. Of each individual infant, only one parent can participate. Parents are excluded if their infant is born prematurely, is diagnosed with a sleep disorder or excessive crying, has a serious medical condition that impedes sleep or wakefulness, receives medication that can influence sleep or wakefulness (e.g. melatonin), or if the infant is part of a multiple birth. Parents are also excluded if they do not speak Dutch at all or receive care from non-regular YHC programs that include sleep advice (e.g. the ‘VoorZorg’ program that provides additional care for parents in vulnerable situations).

**Fig. 1:**
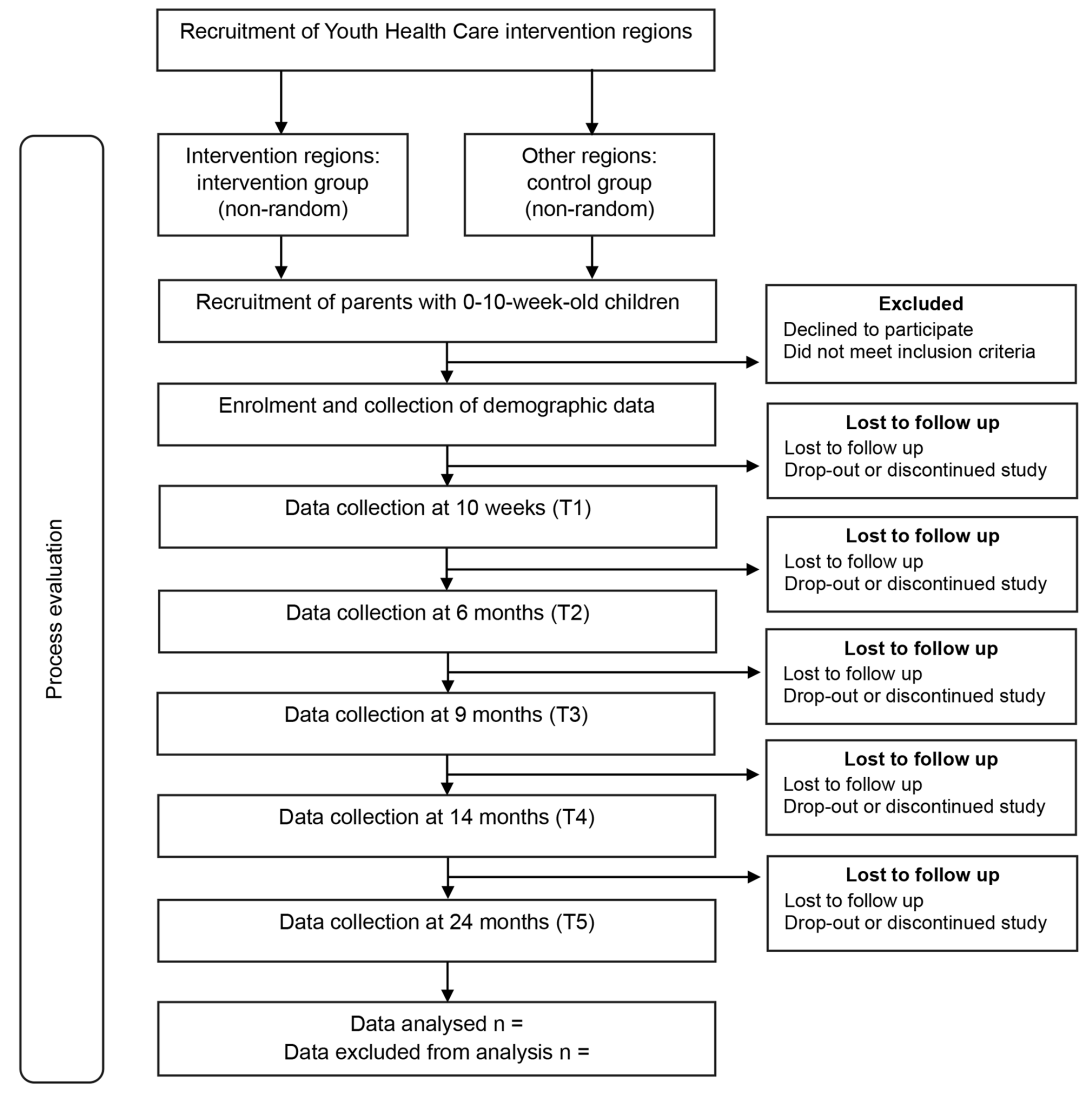
Study flowchart

Multiple strategies will be used to recruit participants. One strategy is to recruit parents via YHC professionals: they will mention the study to parents during YHC contact moments, check for eligibility and hand over the study brochure. Other recruitment strategies include recruiting via regional midwives and maternity care professionals, advertising on social media and visiting locations or organisations parents frequently go to, such as pregnancy classes. Parents receive the (digital) study brochure with which they can access the secured study website that contains the study information, participation criteria and participant information sheet. On the study website parents can also register to join the study and sign the informed consent form. After registration or filling in the informed consent form, all parents are contacted by the research team to check their eligibility. To promote retention and follow-up, participants receive a gift voucher after every completed measurement, up to a cumulative total of €40. See Fig. 1 for the study flowchart.

The sample size calculation is based on the primary outcome measure ‘frequency of nighttime awakenings’, as nighttime awakenings are expected to decrease due to the program and are clinically relevant in improving healthy infant sleep. The effect size is expected to be small to medium (Cohen’s *d* of 0.3). T3 (9 months) is chosen as the main reference timepoint for the primary analysis, as this is the timepoint at which both preventive and behavioural strategies from the program could have been used by parents. Using a conservative estimate based on a one-sided independent samples T-test for two groups, with T3 as the main reference timepoint of the primary analysis, an alpha of 0.05 and a power of 0.80 the total sample size is 278. Assuming a dropout of about 9% the estimated total sample size becomes 306. In addition, to control for potential clustering effects at the level of YHC teams, given an estimated intra-cluster correlation coefficient of about 0.02 and an average cluster size of 4, the total sample size required is 324, with 162 participants in the program group and 162 participants in the control group.

### Program development

The ‘Sleep on number 1’ program was developed and implemented based on continuous iterative co-creation cycles with relevant stakeholders such as parents, health promotion experts and YHC professionals [55, 56]. During this process, the gathered input and feedback was used to develop the program, but also to further improve and adapt the program, support the change process and enhance program implementation [55]. Methods used comprised of qualitative needs assessment interviews with parents and YHC professionals and a discussion session and service design thinking session [57] with YHC professionals, to discuss and validate the interview results and brainstorm about program ideas. Additionally, two consultation groups (one with parents and one with YHC professionals) were created that also provided feedback during program development, adaptation and implementation. During these iterative cycles, the researchers identified the needs and wishes of parents and professionals as well as relevant target behaviours and factors and cognitions influencing these behaviours. The target behaviours included professional’s behaviour in providing sleep education and advice, parental management of infant sleep and parental help-seeking behaviour for infant sleep issues. These behaviours and related factors and cognitions were then matched with evidence-based behavioural change methods [58]. Content-wise, program elements were grounded in evidence-based literature on infant sleep and crying and the corresponding evidence-based Dutch YHC guidelines [53, 54]. These guidelines make a distinction between sleep strategies advised for children below and children older than six months: behavioural strategies that include controlled crying are only considered suitable and effective for children aged six months and older [59].

In conclusion, by using an iterative co-creation approach combined with evidence-based infant sleep health promotion methods, a program was developed that has an evidence-based core and is well-adapted to the local YHC context. Furthermore, the program is somewhat flexible, as the specific evidence-based sleep content of the program can be adapted to fit a certain context. Both YHC program regions received the same program, with some small adaptations to fit the regional context that are described below. The main target groups of the program are parents of 0-2-year-old infants and YHC professionals working with 0-4-year-old infants.

### Program elements targeting youth health care professionals

Firstly, a kick-off symposium was organised for YHC professionals to inform them about the aims of the project, improve their knowledge on infant sleep and conduct the discussion session on the results of the needs assessment interviews. To improve professionals’ sleep knowledge, a researcher and expert on infant sleep (also involved in the development of the Dutch YHC guidelines) gave a presentation about evidence-based prevention and treatment of infant sleep problems. Based on the needs assessment results, in program region 2 also a presentation on inclusive, culture-sensitive health communication was given. In program region 1, the kick-off symposium was held in October 2021 and program region 2 followed in March 2022.

The second program element consisted of the completion of the evidence-based e-learning on infant sleep from the Dutch Centre for Youth Health, to enhance implementation of the YHC guideline on infant sleep [53]. All YHC professionals were invited to complete the e-learning and were allowed to do so during working hours. In program region 1, implementation of the e-learning started in April 2022. Program region 2 started in August 2022. The e-learning had to be completed prior to the skills training described below.

The third program component was a skills training to improve YHC professionals’ knowledge, skills and self-efficacy regarding provision of sleep education and advice. A presentation was given by a researcher and sleep expert on the characteristics of normal infant sleep and the prevention and treatment of infant sleep problems. This was followed by a role-play session in which professionals practiced consultations about infant sleep issues. Role-play was based on fictional cases derived from real life situations. Various components of the YHC guidelines were practiced and elaborated upon, including establishing a diagnosis, using a sleep diary, providing sleep education and developing a tailored treatment plan. Because in program region 2, the Positive Parenting Program is part of their standard YHC working practice, the training given in this region was slightly adapted to fit with this program [60]. In program region 1, the training was held in June and September 2022. In program region 2, the training was held in October 2022.

The fourth program component consisted of two conversation cards about infant sleep: one focussing on infants aged 0–6 months and one on infants aged 6–24 months. The main purpose was to stimulate communication between the parent and professional, structure the conversation and aid in the development of a tailored solution. In addition, a sleep diary and sleep plan were created, to be used together with the conversation cards. The sleep diary comprised of a clear, easy to complete diary for parents to monitor their infant’s sleep. The sleep plan comprised of a structured form for parents to formulate a daily action plan to implement a sleep advice, to increase the chance of successful treatment. Two knowledge clips were made for YHC professionals about the content and use of the conversation cards and related tools. The two conversation cards, sleep diary and sleep plan were developed to be comprehensible for parents, including parents with limited (health) literacy and comprised of maximally B1 language level, according to the Common European Framework of Reference (CEFR) [61]. The conversation card, sleep diary and sleep plan were implemented in program region 1 in September 2022 and in program region 2 in July 2023. The knowledge clips were implemented in February 2023 in program region 1 and in July 2023 in program region 2.

### Program elements targeting parents

A publicly available webpage on the YHC website with information about sleep in 0-2-year-old infants was developed. Parents were referred to the webpage by putting webpage links in the YHC invitation letters and YHC information materials. YHC professionals could also send the link directly to parents. The aim of this webpage was to make evidence-based sleep information available for all parents, irrespective of whether they experienced infant sleep problems or discussed sleep with their YHC professional. The information consisted of the characteristics of normal infant sleep and crying, advice on how to improve infant sleep and behavioural methods to resolve infant sleep problems. The sleep information was developed to be comprehensible for parents with limited (health) literacy and comprised of maximally B1 CEFR language level [61]. A program aim is that the information from the webpage will also be transformed into age-based newsletters that can be send directly to parents (e.g. via e-mail), when YHC information and communication infrastructure allows it in the future. This way, parents can be provided with information that is tailored to their child’s developmental phase. In program region 1, the webpage was implemented in March 2023. In program region 2 the webpage was implemented in July 2023.

### Data collection

#### Primary outcome

**Fig. 2:**
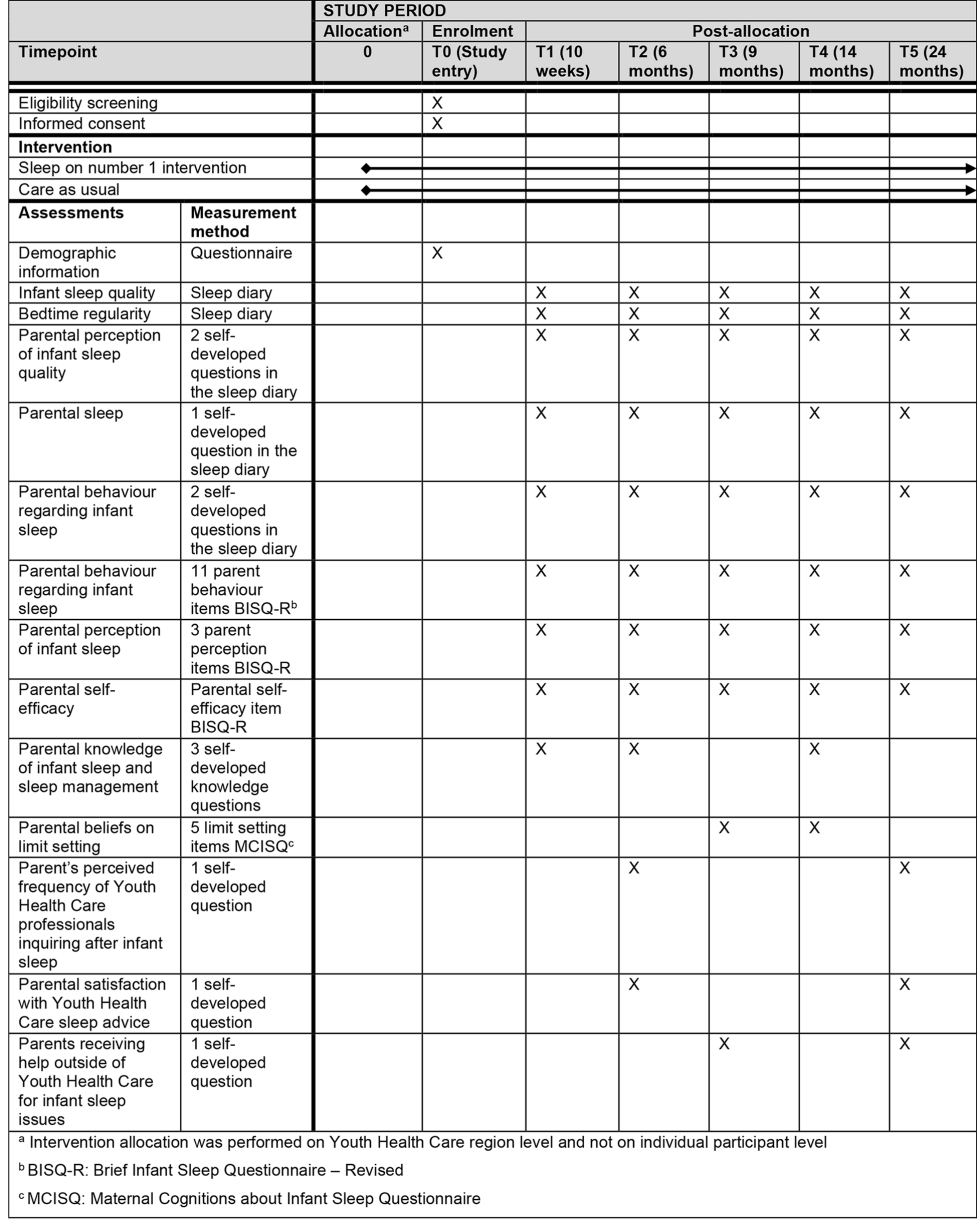
Schedule of enrolment, intervention and assessments for the effectiveness study

The primary outcome of this study is sleep quality of 0-2-year-old infants and will be assessed with a sleep diary filled out by parents, measuring the number and duration of daytime naps, bedtime, time to sleep onset, the number and duration of nighttime awakenings and the time the child gets out of bed in the morning. Parents are asked to fill in the diary for four consecutive days [62]. Sleep quality will be assessed when the infant is 10 weeks, 6 months, 9 months, 14 months and 24 months old. The sleep diary was developed for this study and can be filled in digitally or on paper. All study outcomes, assessment moments and instruments are listed in Fig. 2.

### Secondary outcomes

**Sleep Diary.** Bedtime regularity will be assessed using the bedtime item from the sleep diary. Parental perception of infant sleep quality will be measured with two closed-ended questions integrated in the sleep diary: ‘How well did your child sleep last night?’ and ‘How well did your child sleep today during the day?’ These questions are answered on a five-point Likert scale ranging from very well to very badly. Parental sleep is measured with the question: ‘How did you sleep last night?’ and can be answered on a five-point Likert scale ranging from very well to very badly. Parental behaviour regarding infant sleep management is assessed with two study-developed questions in the sleep diary. The first question focuses on how the parent put the child to bed that night and can be answered with one of the following three answers: ‘awake and not tired’, ‘awake and tired’ and ‘when my child was asleep’. The second questions asks how the child fell asleep that night, and can be answered with one of the following four answers: ‘on his/her own, without a parent in the room’, ‘with a parent present in the room, not attending the child in any way’, ‘with a parent present in the room, attending the child’ or ‘the child fell asleep during feeding’.

**Questionnaires.** The questionnaires of this study can be filled in digitally or on paper. Parental behaviour regarding infant sleep management is assessed with the use of the 11 parental behaviour items from the Brief Infant Sleep Questionnaire (BISQ-R): a validated questionnaire for measurement of infant sleep and related parental behaviours and perceptions [63, 64]. Parental perception of infant sleep and parental self-efficacy regarding infant sleep management are assessed using the three parent perception items and the self-efficacy item from the BISQ-R, respectively.

Parental knowledge of infant sleep will be assessed with three self-developed questions comprising of statements about infant sleep, below which the participant states on a four-point scale whether or not they believe the statement to be true. Knowledge themes examined are normal infant sleep patterns and the expected effects of using a bedtime routine, using a regular, structured day routine and putting the infant to bed at fixed moments. Parental knowledge is measured at 10 weeks, 6 months and 14 months. The knowledge themes at 14 months also include knowledge of behavioural sleep methods, such as controlled crying methods.

Parental beliefs on limit setting are measured with five limit setting items from the validated Maternal Cognitions about Infant Sleep Questionnaire (MCISQ) [65]. Parent’s perceived frequency of YHC professionals inquiring after their infant’s sleep will be investigated with a self-developed question that asks how often YHC professionals generally inquire after their infant’s sleep and can be answered on a five-point Likert scale ranging from always to never.

Parental satisfaction with YHC sleep advice is only investigated in parents that discussed sleep with a YHC professional and is assessed with a self-developed question on how parents perceive YHC sleep advice. The question can be answered on a five-point Likert scale ranging from very good to very bad. Lastly, the number of parents receiving help for infant sleep issues outside of YHC is assessed with a closed-ended question that explores whether parents received help from sources outside of YHC during the prior period. In case parents received help outside of YHC, they are asked to state who provided help.

To be suitable for this study, the used subscales from the BISQ-R and MCISQ were translated into Dutch and were adjusted to be suitable for parents with limited (health) literacy. This was done by three sleep health experts from Maastricht University (the Netherlands) who are also authors of this manuscript, together with an advisor on comprehensible communication and writing for people with limited (health) literacy. The experts are native Dutch speakers also proficient in English. Each expert independently translated the BISQ-R and MCISQ subscales into Dutch versions of these subscales. In a joint session, the three individually translated versions were discussed until consensus was reached on a preliminary Dutch version. Together with the communication advisor, the literacy level of the preliminary Dutch version was adjusted to be maximally B1 CEFR language level. This questionnaire was then translated back to English by another sleep health expert from Maastricht University who is a native English speaker and proficient in Dutch. The original BISQ-R and MCISQ items and the back-translated English versions were then compared on accuracy and clarity. Some final adjustments were made and consensus was reached on the final Dutch versions of the subscales from the BISQ-R and MCISQ.

### Demographic information

In a demographics questionnaire that has to be filled in before the infant is 14 weeks old, parents are asked to provide demographic information about themselves and their infant. Participant’s demographic information consists of their age and family composition and of the gender, ethnicity, educational level and employment status of participant and partner. Infant demographic information consists of infant’s age, gender at birth, birth order position, ethnicity and whether the infant primarily receives breastfeeding, formula feeding or a combination of the two.

### Process evaluation

A mixed method process evaluation will be conducted focusing on evaluation of program reach, adoption, implementation and maintenance and gaining insight in program working mechanisms [49]. The process evaluation will be conducted with parents and YHC professionals (YHC doctors and nurses) from both program regions and comprises of qualitative interviews (partly structured, partly in-depth), quantitative questionnaires and quantitative data regarding program uptake (e.g. the percentage of YHC professionals attending the skills training). The qualitative interviews will be conducted taking into account data saturation and will be audio-recorded with an Olympus voice recorder.

### Data management

The secured website for study registration and informed consent is hosted on the Maastricht University server and managed by the Data and Information Management Centre MEMIC Maastricht University. The personal participant data is stored separately from the study data in the secure Ldot Research Data and Workflow Management System from MEMIC Maastricht University. The sleep diary data is collected with a secure web-based sleep diary from MEMIC Maastricht University and stored in a certified Oracle research database. The certified Castor EDC platform is used for the digital questionnaires and storage of the questionnaire data. To promote retention and complete follow-up, the Ldot Management System assesses at each assessment timepoint whether the participant has filled in the study questionnaire and diary. In case a participant is almost overdue with data entry, a reminder e-mail or text message is sent to the participant, or a phone call is conducted to promote complete follow-up. Participants who fill in the sleep diary and questionnaires on paper, send their anonymous answers to the research team via post. Hereafter, the paper data will be entered in the Castor EDC or Oracle database by the researcher. The original paper documents are safely stored at Maastricht University. All used data management systems are General Data Protection Regulation (GDPR) compliant [66]. Only the research team and involved data management team from MEMIC Maastricht University have access to the study data. To ensure data quality, prior to analysis, all data will be checked by the researcher and will be cleaned to correct for extreme outliers, errors and duplicates.

### Data analysis

Continuous variables will be described in terms of means and standard deviations, while categorical data will be described using percentages and frequencies. Possible demographic differences between the program group and control group will be analysed using independent samples T-tests for continuous outcome variables and Pearson chi-square tests for categorical variables. The primary outcome ‘frequency of nighttime awakenings’ will be analysed by comparing the program group with the control group using a linear mixed model to account for potential confounders or relevant covariates, as well as possible clustering effects at the level of YHC teams, with T3 being the main timepoint of focus of the analysis. In case no substantial clustering effects are found, alternative approaches will be used to analyse the data at T3 if appropriate (e.g. linear regression or a one-sided independent samples T-test).

When appropriate, relevant covariates will be included into the analysis, such as feeding problems and major life events (e.g. parental divorce or illness at the moment of assessment), within a multiple regression analysis framework. In addition to the main analysis, further exploratory analyses will be performed. These include: effectiveness analyses at other assessment times, a repeated measurements analysis to identify changes between groups over the follow-up and analysis of program effects on secondary outcome measures, using the same approach as for the primary outcome in case of continuous variables and logistic regression or chi-square tests in case of categorical variables. All analyses will be performed in IBM SPSS Statistics 27 and/or R. Regarding the process evaluation, quantitative data will be analysed using descriptive statistics. The data of the highly structured part of the qualitative interviews will be reported in frequencies and percentages. The data of the in-depth part of the qualitative interviews will be transcribed verbatim and used as input for the iterative co-creation process and to provide context to the data from the highly structured part.

## Discussion

This paper describes the study protocol for the development, effectiveness and process evaluation of a program tailored to Dutch YHC to promote sleep in 0-2-year-old infants. The program has several strengths. By using continuous, iterative co-creation cycles and methods such as qualitative interviews, a discussion session and a service design thinking session, parents and professionals were involved in program development. This way, a program was created that addresses the needs of important stakeholders and is compatible with the daily working practice of Dutch YHC. This is an important addition to the current work on the promotion of healthy infant sleep, by adding to the limited number of studies that specifically focus on sustainable integration of infant sleep health promotion in practice-based settings such as YHC and by being suitable for parents with limited (health) literacy [30, 32, 67, 68].

Another strength is that the program components provide solutions for various issues considered present in YHC sleep education, by providing training to improve YHC professionals’ knowledge and skills, reducing time constraints with early sleep information provided outside of consultations and by developing program components that are comprehensible for a wide range of parents, including parents with limited (health) literacy [27, 34, 43]. Furthermore, with program elements based on evidence-based behaviour change methods and scientific sleep literature, the program has a sound scientific core and is also adaptable to fit different contexts regarding its specific sleep content. This enhances program implementation and sustainability [44, 46, 58, 69].

Regarding the study design, a strength is that in addition to the main reference timepoint at nine months, both short-term and long-term effects are investigated, due to having five measurement moments, starting at the age of 10 weeks and continuing until the child is two years old. Furthermore, not only infant sleep outcomes, but also intermediary and parental outcomes are assessed, which increases the studies’ sensitivity to find effects and aids in exploring factors that could be related to changes in infant sleep [49, 70]. When evaluating programs in real life settings such as YHC, it is also important to gain insight in program reach, adoption, implementation, maintenance and possible working mechanisms [71, 72]. Therefore, a thorough program evaluation is performed by combining a quasi-experimental effect evaluation with a mixed methods process evaluation [49, 71–73].

This study also has some limitations. One limitation may be that the study relies on subjective parental self-report using a sleep diary for the assessment of infant sleep. However, for this study, self-report measures were considered to be the most suitable option, for they are easy to administer, provide real-time insight in infant sleep and are considered less burdensome for the infant and more acceptable for parents in comparison with wearable sleep monitoring devices [74–76].

Another limitation may be the use of a quasi-experimental post-test only design, which may lower the study’s internal validity. However, for evaluation of complex, practice-based, population-level programs, it is necessary to find the right balance between internal validity and external validity [49, 70, 72, 77]. In the YHC context randomization is difficult, for being a program region requires quite some investment from local professionals, which is not possible in all YHC regions at all times. A post-test only design was chosen because in a study with newborn children, a pre-test measurement cannot be performed in the same child. Hence a quasi-experimental, post-test only study design was determined to be the most suitable design for this study, having the best balance between internal and external validity.

In conclusion, the ‘Sleep on number 1’ program combines the needs and wishes of parents and professionals with evidence-based sleep health promotion and behaviour change methods into a program that is tailored to Dutch YHC. A robust evaluation is ensured by combing a quasi-experimental effect evaluation with a mixed methods process evaluation. If effective, this program has the potential to be implemented in and disseminated across YHC regions to sustainably promote healthy infant sleep on a population level [30, 34, 35, 38].

### Electronic supplementary material

Below is the link to the electronic supplementary material.

Additional file 1: Completed SPIRIT checklist

## Data Availability

No datasets were generated or analysed during the current study.

## Abbreviations

BISQ-R: Brief Infant Sleep Questionnaire – Revised
CEFR: Common European Framework of Reference
GDPR: General Data Protection Regulation
MCISQ: Maternal Cognitions about Infant Sleep Questionnaire
RE-AIM: Reach, Effectiveness, Adoption, Implementation and Maintenance
SEP: Socioeconomic Position
SPIRIT: Standard Protocol Items: Recommendations for Intervention Trials
YHC: Youth Health Care
